# Special Issue “Applications of Porous Nanotubes”

**DOI:** 10.3390/ma15144833

**Published:** 2022-07-11

**Authors:** Daniil Eurov, Dmitry Kurdyukov

**Affiliations:** Ioffe Institute, 195256 St. Petersburg, Russia; kurd.gvg@mail.ioffe.ru

“Applications of Porous Nanotubes” is an open Special Issue of *Materials*, which aims to publish original contributions and review papers on new fundamental and applied aspects of porous nanomaterials, and to investigate their properties and underlying mechanisms for development of various porous media with a desired functionality.

Nanomaterials are of great interest due to their specific and fascinating properties, which are superior to their bulk counterparts. Porous nanotubes are a promising type of nanomaterials with exceptional chemical and physical characteristics, owing to their unique quasi one-dimensional geometric features, which makes them of particular interest. Carbon-based nanotube variants were the first of this class of materials [1]. Single- or multicomponent porous nanotubes made of various materials such as metals, metal oxides, nitrides, chalcogenides, etc. have gained much attention in the past couple of decades, since they possess enhanced sensing ability, catalytic activity, and photovoltaic behavior. For instance, metal-oxide nanotubes can be used for a wide range of applications, such as selective biological and chemical sensors, solar cells and photocatalysts [2].

There are two main approaches to obtaining nanotubes: directed (soft and hard template) and non-directed (self-organization) methods. When developing a synthetic method for the fabrication and/or functionalization of porous nanotubes, the most important issue that one needs to address is the simultaneous control over dimensions, morphology, monodispersity, and chemical purity. These characteristics largely determine potential applications of nanotubes as catalysts, gas sensors, solar cells, supercapacitors, biological sensors, carriers for drug delivery, etc. In particular, different synthetic conditions of Ti-based nanotubes, which are promising as effective medical implants, may strongly affect their properties such as biocompatibility, and resistance to bio-corrosion, which makes the development of proper technological routes a complex and important issue [3].

The research interest of the section “Applications of Porous Nanotubes” includes various topics, ranging from, but not limited to, the fabrication of nanotubes of various composition using versatile synthetic approaches and their application in sensing, catalysis, energy harvesting, electronic devices, and biomedicine.
